# Clinical characteristics and outcomes of atrial fibrillation and flutter at the Aga Khan University Hospital, Nairobi

**DOI:** 10.5830/CVJA-2012-064

**Published:** 2013-03

**Authors:** Jay Shavadia, Gerald Yonga, Sitna Mwanzi, Ashna Jinah, Abednego Moriasi, Harun Otieno

**Affiliations:** Department of Cardiology, Aga Khan University Hospital, Nairobi, Kenya; Department of Cardiology, Aga Khan University Hospital, Nairobi, Kenya; Department of Cardiology, Aga Khan University Hospital, Nairobi, Kenya; Department of Cardiology, Aga Khan University Hospital, Nairobi, Kenya; Department of Cardiology, Aga Khan University Hospital, Nairobi, Kenya; Department of Cardiology, Aga Khan University Hospital, Nairobi, Kenya

**Keywords:** atrial fibrillation, clinical characteristics, Kenya, outcomes

## Abstract

**Introduction:**

Scant data exist on the epidemiology and clinical characteristics of atrial fibrillation in Kenya. Traditionally, atrial fibrillation (AF) in sub-Saharan Africa is as a result of rheumatic valve disease. However, with the economic transition in sub-Saharan Africa, risk factors and associated complications of this arrhythmia are likely to change.

**Methods:**

A retrospective observational survey was carried out between January 2008 and December 2010. Patients with a discharge diagnosis of either atrial fibrillation or flutter were included for analysis. The data-collection tool included clinical presentation, risk factors and management strategy. Follow-up data were obtained from the patients’ medical records six months after the index presentation.

**Results:**

One hundred and sixty-two patients were recruited (mean age 67 ± 17 years, males 56%). The distribution was paroxysmal (40%), persistent (20%) and permanent AF (40%). Associated co-morbidities included hypertension (68%), heart failure (38%) diabetes mellitus (33%) and valvular abnormalities (12%). One-third presented with palpitations, dizziness or syncope and 15% with a thromboembolic complication as the index AF presentation. Rate-control strategies were administered to 78% of the patients, with beta-blockers and digoxin more commonly prescribed. Seventy-seven per cent had a CHA_2_DS_2_VASC score ≥ 2, but one-quarter did not receive any form of oral anticoagulation. At the six-month follow up, 6% had died and 12% had been re-admitted at least once. Of the high-stroke risk patients on anticoagulation, just over one-half were adequately anticoagulated.

**Conclusion:**

Hypertension and diabetes mellitus, not rheumatic valve disease were the more common co-morbidities. Stroke risk stratification and prevention needs to be emphasised and appropriately managed.

## Abstract

In developed countries, atrial fibrillation is the most common sustained rhythm disorder, with prevalence increasing with age.^1^,^2^ This rhythm disorder is associated with mortality and significant morbidity due to increased stroke risk, heart failure, hospitalisations and reduced quality of life.^3^-^5^ Data from other parts of Africa support the notion of the ‘double burden of disease’, grappling with increasing cardiovascular disease in addition to the existing maternal and child health problems and infectious disease burden.^6^

Atrial fibrillation, the global arrhythmia epidemic, is proposed to have a more severe epidemiology in Africa, with the incident age being relatively younger and attendant complications more prevalent,^7^,^8^ possibly due to a combination of rheumatic valve disease burden and non-adherence to established clinical guidelines. The purpose of this study was to obtain the epidemiology, predisposing factors, clinical presentation and outcomes of atrial fibrillation (AF) and atrial flutter (AFL) at a private urban referral teaching hospital in East Africa.

## Methods

This retrospective survey was performed at the Aga Khan University teaching hospital in Nairobi, Kenya, between January 2008 and December 2010. The Aga Khan University Hospital is a 256-bed hospital serving predominantly an urban middle- to high-income community.

Patients over the age of 18 years, who had an electrocardiographic (ECG) diagnosis of either AF or AFL during their admission were included in the study. The data were collected using a data tool that included patient demographics and clinical presentation, potential risk factors to the development of AF and AFL, management strategy, and anticoagulation status. Follow-up data were from the patients’ medical records obtained six months after the index presentation.

Atrial fibrillation was sub-divided into three main classes: paroxysmal (initial or recurrent AF episodes terminating spontaneously within seven days), persistent (non-spontaneously terminating after seven days or requiring electrical or chemical cardioversion), and permanent AF (failed rhythm control with electrical and chemical cardioversion, or cardioversion had never been attempted). ‘Lone AF’ was defined as an episode in patients less than 65 years of age, and without structural cardiac abnormalities. Patients who had not completed 12 months since the index AF event at the six-month follow up were classified as having ‘incomplete follow up’ for the purposes of our study.

We used the CHADS2 scoring system^9^ to stratify patients for prediction of thrombo-embolic (TE) and stroke risk. This scheme has been validated, although not in African patients, to provide a predictive value of TE risk. A score of ≥ 2 predicts a significant TE risk, warranting anticoagulation, while a score of 0 or 1 predicts moderate risk, favouring anticoagulation over aspirin.

A comprehensive two-dimensional transthoracic echocardiography with pulsed- and continuous-wave Doppler and colour-flow velocity spectral imaging was performed to determine the severity of valvular heart disease in only patients with clinical signs suggestive of valvular heart disease. Patients with echocardiographic moderate to severe mitral or aortic stenosis, or moderate to severe aortic and mitral regurgitation were classified as having valvular heart disease. Patients with either mitral stenosis, or combined mitral stenosis and aortic regurgitation were labelled as having rheumatic heart disease (RHD).

All continuous variables are expressed as mean ± standard deviation. Categorical variables are expressed as percentages.

## Results

In this survey, 162 patients from 22 144 general hospital medical admissions were recruited over a 36-month period. Their baseline characteristics are given in Table 1. Ninety-five per cent of the patients recruited had AF, with the rest having AFL. The mean age at presentation was 67 years, with incidence increasing with age and peaking at the age bracket 70–100 years, as described in Table 1.

**Table 1:** Baseline Characteristics

Mean age (years)	67.8 ± 17.1
Incidence by age bracket (years )	
18–30 (%)	3.1
31–50 (%)	13.0
51–70 (%)	26.9
71–100 (%)	57.0
Race	
Native Africans	46.8
Asians	30.7
Caucasians	22.5
AF:AFL	19:1
Male: female	1.27:1
SBP at diagnosis (mmHg)	131 ± 28
DBP at diagnosis (mmHg)	78 ± 16
Heart rate at diagnosis	95 ± 35
BMI	27.2 ± 5.8
AF subtype	
Paroxysmal (%)	40
Persistent (%)	13.5
Permanent (%)	40
Incomplete follow up (%)	6.5
Reason for presentation	
AF/AFL (%)	32.1
Heart failure (%)	17.3
TE event (%)	15.5
Sepsis (%)	13.6
Other (%)	21.5
Risk factors	
Hypertension (%)	68
Heart failure (%)	38
Diabetes mellitus (%)	33
Coronary artery disease (%)	19
Valvular heart disease (%)	12

SBP: systolic blood pressure, DBP: diastolic blood pressure, TE: thrombo-embolic event.

In terms of haemodynamics at presentation, 5% presented with hypotension (systolic blood pressure: SBP ≤ 90 mmHg), and 46% with a rapid heart rate (resting heart rate ≥ 90 beats/min). Thirty-two per cent of the patients presented to hospital due to symptoms related to their rapid heart rate (palpitations, dizziness, syncope and fatigue), 17% had congestive heart failure, 15% thrombo-embolic events (transient ischaemic attack, cerebrovascular accident, other embolic events), 8.3% for other surgical indications, and 1.9% due to acute coronary syndrome and major bleeding, respectively.

Hypertension (68%), heart failure (38%), diabetes mellitus (33%) and coronary artery disease (19%) were the commoner underlying predisposing factors; valvular heart disease (12%), chronic obstructive airway disease (7%), excess alcohol intake (5%) and hyperthyroidism (3%) accounted for the other predisposing risk factors of atrial fibrillation. Only six (32%) of the 19 patients who had valvular heart disease had echocardiographic evidence of rheumatic heart disease.

Rate control was the more preferred strategy for management of arrhythmia (78.4%), while the remainder were managed with a rhythm-control approach. The choice of both rate- and rhythmcontrol agents is summarised in Table 2. Amiodarone was the only agent used for chemical cardioversion, while direct-current cardioversion was opted for in 37.1% of the patients in the rhythm strategy. AF ablation was not performed in any of the patients, as this modality of rhythm control is not locally available.

**Table 2:** Rhythm Management Strategy

*Management strategy*	*Rate control (%) (n = 127/162)*	*Rhythm control (%) (n = 35/162)*
Digoxin alone	38 (29.9)	–
BB alone	36 (28.3)	–
BB + digoxin	32 (25.2)	–
CCB	10 (7.8)	–
Amiodarone alone	3 (2.3)	9 (25.8)
BB + amiodarone	4 (3.1)	–
BB + CCB	2 (1.7)	–
CCB + digoxin	2 (1.7)	–
Spontaneous cardioversion	–	13 (37.1)
DC cardioversion	–	13 (37.1)

BB: beta-blockade, CCB: non-dihydropyridine calcium channel blockers, DC: direct current.

For stroke risk categorisation, 18.6, 16.7 and 64.7% of the patients had a CHADS_2_ score of 0, 1 and ≥ 2, respectively. Of the patients with a CHADS_2_ score ≥ 2, 21.2% did not receive any form of anticoagulation, with the majority being on aspirin. Of the patients with a CHADS_2_ score between 0 and 1, 36.4% were on aspirin, 29.1% on oral anticoagulation and 34.5% on no stroke-prevention strategy (Table 3).

**Table 3:** Stroke Risk Stratification And Management

CHADS_2_ score	0	1	≥ 2
Total number	29/156 (18.6)	26/156 (16.7)	101/156 (64.7)
No. on anticoagulation (%)	5 (4.4)	11 (40)	80 (79.8)
No. on ASA (%)	13 (47.8)	7 (40)	17 (17.8)
No. on DAT (%)	0	0	2 (1.6)
No. on no treatment (%)	11 (47.8)	8 (20)	2 (0.8)

ASA: aspirin, DAT: dual antiplatelet therapy.

Follow-up data six months after the patients’ index presentations were available for 124 of the 162 patients (76.5%) recruited to the study. Of these, eight had died, and the cause of death was sepsis related in three, cardiovascular in two and unclear in the rest. Fifteen patients had made at least one hospital revisit, 11 of whom had been re-admitted. Five of these were due to decompensation of heart failure, four due to major bleeding, and one each due to an acute coronary syndrome and an acute ischaemic stroke, respectively. Seven patients had reported an accidental fall at home and this accounted for two of the four patients admitted with a major bleed. At the six-month follow up, 100 of 124 (80.6%) patients were on a rate-control strategy, while the remaining 24 were on a rhythm-control strategy (19 amiodarone, three flecainide, one propafenone, one sotalol).

## Discussion

Epidemiologically, the results of this survey were relatively similar to a study from Cameroon,^7^ with the mean age of patients being in the mid-sixties. There was a higher preponderance of males in our study. A dominance of native Africans was noted, followed by Asian and Caucasian races. However, the majority of the Asians and Caucasians in our study had been long-term residents in Kenya.

As in the developed countries, AF prevalence in Africa increases with increasing age. Over 80% of our patients were over 50 years of age, and more than 50% of the total study population were in the over-70-year age bracket. This observation was also reflected in the data from the Heart of Soweto study,^8^ where a steep increase in case presentation was noted after 50 years of age.

Consistent with existing data, the relationship between hypertension and the development of AF has been traditionally documented, and is thought to be due to changes in myocyte ultrastructure and physiology.^10^ In our population, despite the relatively high background prevalence of RHD,^11^,^12^ hypertension was present in over two-thirds of our AF patients. This supports the notion of aggressive screening and treatment for hypertension, as this is singled out as the most common modifiable risk factor driving this arrhythmia epidemic, even in developing countries. Uncontrolled hypertension then progresses to hypertensive heart disease and heart failure, which again was the next most common predisposition, present in over one-third of our patients.

There are no local guidelines and protocols available for the management of AF, and treating physicians would decide on a rate- or rhythm-control approach, based on either their preference or on international guidelines. Due to the pro-arrhythmogenicity of anti-arrhythmic agents, their inappropriate usage may account for an increased mortality rate of AF in our setting.

It was surprising to note that 15% of patients presented with a TE event as the index presentation of AF. It was even more surprising that one in every five patients who needed to be on anticoagulation was on aspirin, an aspirin–clopidogrel combination or no treatment at all. With a five-fold increased stroke risk with AF, our data not only serves to highlight the gravity of the morbidity associated with undiagnosed AF, but more importantly, inappropriate stroke risk stratification by the treating physicians.

Use of the CHA_2_DS_2_VASC scoring system^13^ may improve the predictive index of a TE event, especially in the CHADS2 score 0–1 cohort. However, its non-validation in the native African population currently limits its utility in our study setting.

Prevalence of the disease in the elderly and the risk of major bleeding with oral anticoagulation form a sinister recipe for complications in AF. This was highlighted by accidental falls reported in 5.6% of patients, and re-admissions for major bleeding in 3.2% of patients. In an environment with poor infrastructure and emergency medical services, physician choice of antiplatelets over oral anticoagulants, even in patients needing anticoagulation may be understandable.

The study site, being a private teaching hospital, may have introduced a sampling bias, hence the significantly low proportion of patients with rheumatic valvular disease in our cohort. Additionally, performing echocardiography for only patients who had clinical evidence of RHD may also have accounted for the low numbers of valvular AF in our study, despite a significant background prevalence of RHD.

Our study had several other limitations. (1) Due to the retrospective design, classification of AF subtypes had to be made on available medical records. For patients presenting in the last six months of the study, an inadequate length of follow up prevented appropriate categorisation of these patients. (2) An echocardiographic evaluation was not available for all patients. This would have provided supplementary data on the presence and severity of structural heart disease, and assist in better characterising both valvular and non-valvular AF. (3) A longer follow-up period to assess for hard end-point complications of AF was not possible due to patients returning for medical care only when severely ill.

## Conclusion

Clinical characteristics of AF in Kenya are similar to data from other parts of Africa. However, non-valvular AF is more predominant in our setting, with hypertension, heart failure and diabetes being the most common associated co-morbidities. A rate-control modality using digoxin and beta-blockade is the predominant treatment strategy. Stroke risk assessment and stratification is sub-optimally performed in Kenya, with a significant proportion of eligible patients not receiving anticoagulant therapy. Regional data on risk factors and complications of AF should be pooled to generate locally tailored guidelines in an attempt to achieve better adherence to the provision of AF care.
